# Development of White Cabbage, Coffee, and Red Onion Extracts as Natural Phosphodiesterase-4B (PDE4B) Inhibitors for Cognitive Dysfunction: *In Vitro* and *In Silico* Studies

**DOI:** 10.1155/2024/1230239

**Published:** 2024-05-21

**Authors:** Nazir Ahmad, Kaisun Nesa Lesa, Navista Sri Octa Ujiantari, Ari Sudarmanto, Nanang Fakhrudin, Zullies Ikawati

**Affiliations:** ^1^Department of Pharmacology and Clinical Pharmacy, Faculty of Pharmacy, Universitas Gadjah Mada, Sekip Utara, Yogyakarta 55281, Indonesia; ^2^Department of Food and Nutritional Science, Khulna City Corporation Women's College, Affiliated to Khulna University, Khulna, Bangladesh; ^3^Department of Food and Agricultural Product Technology, Faculty of Agricultural Technology, Universitas Gadjah Mada, Yogyakarta 55281, Indonesia; ^4^Department of Pediatrics, Nihon University Hospital, Tokyo, Japan; ^5^Department of Nutrition and Food Technology, Jessore University of Science and Technology, Jessore, Bangladesh; ^6^Department of Pharmaceutical Chemistry, Faculty of Pharmacy, Universitas Gadjah Mada, Sekip Utara, Yogyakarta 55281, Indonesia; ^7^Department of Pharmaceutical Biology, Faculty of Pharmacy, Universitas Gadjah Mada, Sekip Utara, Yogyakarta 55281, Indonesia; ^8^Medicinal Plants and Natural Products Research Center, Faculty of Pharmacy, Universitas Gadjah Mada, Sekip Utara, Sleman 55281, Yogyakarta, Indonesia

## Abstract

Human cognition fundamentally depends on memory. Alzheimer's disease exhibits a strong correlation with a decline in this factor. Phosphodiesterase-4 B (PDE4B) plays a crucial role in neurodegenerative disorders, and its inhibition is one of the promising approaches for memory enhancement. This study aimed to identify secondary metabolites in white cabbage, coffee, and red onion extracts and identify their molecular interaction with PDE4B by *in silico* and *in vitro* experiments. Crushed white cabbage and red onion were macerated separately with ethanol to yield respective extracts, and ground coffee was boiled with water to produce aqueous extract. Thin layer chromatography (TLC)–densitometry was used to examine the phytochemicals present in white cabbage, coffee, and red onion extracts. Molecular docking studies were performed to know the interaction of test compounds with PDE4B. TLC-densitometry analysis showed that chlorogenic acid and quercetin were detected as major compounds in coffee and red onion extracts, respectively. *In silico* studies revealed that alpha-tocopherol (binding free energy (∆*G*_bind_) = −38.00 kcal/mol) has the strongest interaction with PDE4B whereas chlorogenic acid (∆*G*_bind_ = −21.50 kcal/mol) and quercetin (∆*G*_bind_ = −17.25 kcal/mol) exhibited moderate interaction. *In vitro* assay showed that the combination extracts (cabbage, coffee, and red onion) had a stronger activity (half-maximal inhibitory concentration (IC_50_) = 0.12 ± 0.03 *µ*M) than combination standards (sinigrin, chlorogenic acid, and quercetin) (IC_50_ = 0.17 ± 0.03 *µ*M) and rolipram (IC_50_ = 0.15 ± 0.008 *µ*M). Thus, the combination extracts are a promising cognitive enhancer by blocking PDE4B activity.

## 1. Introduction

Cognitive dysfunction is a serious health issue worldwide; this condition is primarily caused by Alzheimer's disease (AD), which is characterized by the aggregation of amyloid-*β* plaques and neurofibrillary tangles of hyperphosphorylated tau proteins [1]. In 2020, dementia was estimated to affect 55 million individuals globally [2]. The declining concentrations of cyclic adenosine monophosphate (cAMP), cyclic guanosine monophosphate (cGMP), and brain-derived neurotrophic factor (BDNF) in the hippocampus have been observed to be attributed to AD development [1]. The exploration of nootropics or cognitive enhancers, known as “smart drugs,” is catching intrigue on a daily basis. Healthy participants (students and workers) generally take smart drugs to enhance their memory, attention, learning, executive functions, and vigilance. The benefits and potential risks are associated with the use of the most common synthetic nootropics in healthy adults, including piracetam, benzodiazepine inverse agonists, methylphenidate, donepezil, amphetamine-type stimulants, and unifiram analogues [3–5]. Nootropics that target phosphodiesterases (PDEs) in the brain aid in the normal regulation of neuronal cells [6–8]. To date, the world is paying more attention to cognitive enhancement using natural sources, but the research on the effects of white cabbage, coffee, and red onion as smart drugs is still unavailable [9, 10].

Phosphodiesterase-4 (PDE4), the largest family of PDE, is the most complicated and maybe the most broadly expressed PDE in the body [11], and it particularly hydrolyzes cAMP into its inactive form, 5-AMP [12]. Phosphodiesterase-4B (PDE4B) is strongly expressed in the *cornu ammonis* 2 and *cornu ammonis* 3 areas of the hippocampus, the cerebellar granular layer, piriform, and parietal cortex [13]. The continuous rise in the production of PDE4 has been observed in the brains of AD patients, specifically those with hippocampal dysfunction and cognitive impairment [14–16]. PDE4 inhibitors (first generation), such as rolipram ameliorate cognition, mitigate despair-like behaviors, anxiety, and promote neuroplasticity in the hippocampus subsequent to transient global cerebral ischemia [17], and they also exhibited serious side effects (nausea and emesis) at therapeutic doses. In similar manner, second-generation PDE4 inhibitors, including roflumilast and cilomilast, augment memory and cognition, increase neurogenesis, and promote neuroplasticity of the hippocampus [18]. In clinical trials, the maximal dose remained limited owing to adverse effects [19]. For the resolution of this problem, an alternative PDE4B inhibitor, specifically one that originates from natural sources (plants), is required. According to the previous studies, dichloromethane *Uvaria alba* (*U. alba*) subextract was used to target PDE4 B2B and acetylcholinesterase (AChE) in *in vitro* assay due to its neuroprotective properties, especially in AD for the discovery of drugs [20]. The flavonol-enriched n-butanol fraction of *U. alba* attenuated lipopolysaccharide-induced inflammatory responses through the inhibition of proinflammatory NF-*κ*B pathway and the increased NRF_2_ levels [21] that are beneficial in neuroprotection. Moreover, in *in vitro* assay, sappanone A (obtained from heartwood of *Caesalpinia sappan* L.) effectively suppressed PDE4B1 enzyme activity and decreased TNF-*α* production in RAW264.7 macrophages leading to dual antioxidant and anti-inflammatory activities [22]. Vascular dementia, which makes up around 20% of dementia cases, is the second most prevalent type of senile dementia after AD. For vascular dementia, no medications are currently licensed. A wide range of risk factors, such as cerebral hemorrhage, cerebral infarct, arterial hypotension, and cerebrovascular disorders, have been linked to vascular dementia, even if the exact cause of the condition is still unknown. Moreover, changes in cyclic nucleotide levels, neuroinflammation, and excitotoxicity could all be part of the underlying mechanism. In a recent study, twenty-five *α*-mangostin derivatives from *Garcinia mangostana*, administered at a dose of 10 mg/kg, showed impressive therapeutic effects in a vascular dementia model and spared beagle dogs from vomiting, suggesting that these derivatives have potential as a novel antivascular dementia drug [23]. Therefore, white cabbage, red onion, and coffee extracts were considered for this study to find a natural cognitive enhancer.

White cabbage (*Brassica oleracea*), family Brassicaceae, is consumed worldwide both as a vegetable and in traditional medicine [24]. This vegetable has been investigated for antipsychotic [25], anticholinesterase [26], anti-inflammatory [27, 28], and antioxidant activities [29]. White cabbage contains abundant amounts of alkenyl glucosinolate, also known as sinigrin. Previously, sinigrin has been shown to increase the levels of cyclic nucleotides (cAMP) by blocking PDE4 in the lungs [30]. On the flip side, coffee (*Coffea robusta*), family Rubiaceae, is consumed as a beverage, and because of its chlorogenic acid presence, effervescent granules are used as an antidiabetic agent [31, 32] as well as often preferred because of its alertness enhancing, drowsiness, and fatigue-removing properties [33]. Coffee has a wide range of pharmacological applications [31, 32], including anti-Parkinson, anti-Alzheimer, and memory enhancement, and reduces the incidence of dementia [33, 34], antiamyloidogenic [35], anti-inflammatory [36], antioxidant [37, 38], and antiglycation activities [38]. Its phytochemical components, such as caffeine and chlorogenic acid, are linked to these effects [33]. *C. robusta* is also a rich source of hydroxycinnamic acid derivatives, such as chlorogenic acid [39]. Chlorogenic acid and its products can cross the blood-brain barrier (BBB) and offer neuroprotection. In addition, chlorogenic acid has been studied for its potential to boost memory in those who have memory deficiencies caused by sleep deprivation by inhibiting PDE4 activity [40]. On the other hand, red onion (*Allium caepa*), family Amaryllidaceae, is an extensively consumed vegetable [41]. *A. cepa* has shown some reported biological activities, namely, antioxidant [42], anti-inflammatory [43], neuroprotective properties [44, 45], and its flavonoids help to reduce the risk of dementia [46]. *A. cepa* is a complete source of beneficial bioactive compounds, especially flavonoids [47]; among them, quercetin is a major phytochemical [48] that can penetrate BBB, inhibit PDE4, and promote the activation of kinases (such as protein kinase A). These kinases phosphorylate the transcriptional factor cAMP-responsive element-binding protein (CREB). The CREB induces the expression of genes for neuronal plasticity, offers neuroprotection, and improves memory function [46]. Different components [49] or combinations of herbs function synergistically and are, therefore, an essential part of their therapeutic efficacy; many of the phytomedicines on the drug market are complete extracts of plants [50, 51]. The three aforementioned were selected for this study because their major compounds (chlorogenic acid, quercetin, and sinigrin) have been previously shown to interact with PDE4. Furthermore, because these plants are safe and widely accessible, further research into their effects, either alone or in combination, can be done in vitro and in silico to increase PDE4B inhibition.

There is currently no evidence, either *in vitro* or *in silico*, that white cabbage, coffee, and red onion extracts as nootropics alone or in combination inhibit PDE4B and identify the chief bioactive chemicals present in coffee (in aqueous), red onion, and white cabbage (in ethanol) extracts by thin layer chromatography (TLC)–densitometry has been conducted. This investigation aimed to identify the major bioactive compounds in white cabbage, coffee, and red onion and to evaluate their activity against PDE4B via *in silico* and *in vitro* studies as well as computational pharmacokinetics and toxicities prediction.

## 2. Materials and Methods

### 2.1. Materials

Fresh white cabbage (Magelang District, Middle Java, Indonesia), roasted coffee seeds (Lampung, South Sumatra, Indonesia), fresh red onion (Magelang District, Middle Java, Indonesia), deionized water (PT Bratachem, Yogyakarta, Indonesia), 0.9% saline (PT Braun Pharmaceutical Indonesia), aquabidest, ethanol (Sigma-Aldrich, USA), 1,4-dithiothreitol (Sigma-Aldrich, USA), Whatman no. 1 (filter paper) (GE HealthCare, USA), silica-gel F254 (20 cm × 20 cm) (Merck, Germany), sinigrin (Sigma-Aldrich, USA), chlorogenic acid (Sigma-Aldrich, USA), quercetin (Merck, Germany), ethyl acetate (Merck, Germany), formic acid (Merck, Germany), n-Hexane (Merck), acetone (Merck, Germany), toluene (Merck, Germany) and chloroform (Merck, Germany), sprays (FeCl3 and citro borate) (Merck, Germany), PDE4B1 assay kit (BPS Bioscience, USA), test ligands (Table 1; total = 61 compounds), PDE4B_HUMAN (4KP6) enzyme (UniProt ID: Q07343), known ligands (total = 1863), and Molecular Operating Environment (MOE) software (MOE 2022.10) were used. CAMAG® TLC Scanner 3 (Muttenz, Switzerland) and Spark® 20M Multimode Reader Tecan (Switzerland) were also employed in this research.

### 2.2. Collection and Identification of Plant Material

White cabbage and red onion (fresh whole bulb) were collected from Magelang, Jawa Tengah, and coffee seeds were purchased from Tokopedia (Lampung origin). All these test materials were authenticated at the Department of Pharmaceutical Biology, Faculty of Pharmacy, Universitas Gadjah Mada, Yogyakarta, Indonesia (Identification no. 20.25.1 UN1/FFA.2/BF/PT/2022).

### 2.3. Preparation of Extracts

White cabbage (1 kg) and red onion bulb (1 kg) were rinsed with water and chopped separately. The chopped materials were later placed in an electrical blender one by one and processed to obtain a finer form for extraction. Roasted coffee (1 kg) was also blended with an electrical blender. The blended samples (white cabbage and red onion) were macerated with pure ethanol 100% (percent) (5 L) for each sample and placed overnight for at least 24 hours. The coffee was macerated by the infusion method, where ground coffee was boiled in distilled water for 15 minutes. Whatman No. 1 filter paper was used to filter all extracts, and the solvents were evaporated using a rotary evaporator at 50–70°C, and the extracts were stored in a desiccator at room temperature until dryness.

### 2.4. TLC and TLC-Densitometry Analysis

TLC analysis was performed on an aluminum plate precoated with silica-gel F254 as the stationary phase and the mixtures of formic acid, ethyl acetate, and aquabidest (1 : 8 : 1.5; 25) and n-hexane, ethyl acetate, and formic acid (6 : 4 : 0.5; 19) as a mobile phase for coffee and red onion, respectively. To visualize the compounds, the TLC spots were observed under a ultraviolet (UV) light at 254 and 366 nm wavelength, as well as with FeCl_3_ and citroborate for chlorogenic acid and quercetin, respectively. TLC-densitometry analysis was used to identify chlorogenic acid (1 mg/mL) and quercetin (1 mg/mL) in coffee (10 mg/mL) and red onion (10 mg/mL) extracts solution, respectively. The maximum wavelengths of 330 nm and 380 nm were set for the TLC-densitometry analysis of chlorogenic acid and quercetin, respectively.

### 2.5. *In Silico* Molecular Docking Studies

To determine the binding interaction of the test ligand with PDE4B (4KP6), molecular docking was carried out using the Molecular Operating Environment (MOE) software (MOE 2022.10), and the majority of the default tools (pocket atom, placement method (Triangle Matchter, London dG), refinement method (Induced fit and ASE), and energy minimization as 0.1 kcal/mol) were followed. Protein Data Bank (RCSB PDB, https://www.rcsb.org/) was used to acquire protein crystals of the PDE4B_HUMAN enzyme (UniProt ID: Q07343). For the desired expression, only 4KP6 was selected for docking, and the two-dimensional structures of previously investigated compounds were attained using SMILES ID in PubChem (https://www.pubchem.ncbi.nlm.nih.gov/).

#### 2.5.1. Preparation of Known Ligand Dataset

The dataset of all known ligands (inhibitors of PDE4B; total = 1863 known ligands) for PDE4B, which was selected based on the ligands' activity (half-maximal inhibitory concentration (IC_50_)), was downloaded from the ZINC database (https://zinc.docking.org/). Subsequently, the known ligand dataset was created using MOE's default parameters following minimizing energy by MOPAC in MOE (0.1 kcal/mol).

#### 2.5.2. Preparation of Test Ligands

All test ligands' 3D structures (of total = 61 bioactive compounds from white cabbage, coffee, and red onion; Table 1) were created by MOE by minimizing energy following minimizing energy by MOPAC in MOE (0.1 kcal/mol) and saving them in a (.mdb) file, and their 2D structures were retrieved from their smiles ID in PubChem.

#### 2.5.3. Preparation of Protein-Ligand Complex

A total of forty-two protein (PDE4B) crystal structures (including 4KP6) were retrieved from the PDB. Using quickprep in MOE software with the default parameters, the 3D structure of PDE4B (4KP6) was prepared by eliminating metal atoms and minimizing energy by MOPAC in MOE (0.1 kcal/mol). The crystal protein 4KP6 complex was then saved as an MOE file for further docking validation.

#### 2.5.4. Validation of Docking Protocols

To validate the pose or placement algorithm of the docked ligand, 4KP6 was redocked (by applying Induced fit, London dG, and ASE protocols) with its native ligand. Redocking and scoring function validation were used to validate the docking protocol. The flexible docking on the protein's pocket atoms was done using the induced fit method. The placement method was chosen as Triangle Matcher and London dG, and the scoring function was chosen as ASE. Before molecular docking of the test ligands, pose validation and scoring function of known ligands were analyzed. To evaluate pose validation, the binding affinities for ligand-enzyme complexes were calculated as kcal/mol, and the root mean square deviation (RMSD) was computed. The RMSD value was regarded to be within the threshold limit, <2 Å (angstrom). The association between docking score and IC_50_ values of recognized ligands was calculated in order to examine the scoring function validation.

#### 2.5.5. Docking of Test Ligands with Protein

All the prepared 61 test ligands (excluding their six repetitions of the same compounds in the table) from white cabbage, coffee, and red onion (Table 1) were docked with the prepared PDE4B (4KP6) using the Induced fit, London dG, and ASE protocols, with 10 poses being set for each component.

### 2.6. Profiles of ADME

SwissADME software (Molecular Modeling Group, Swiss Institute of Bioinformatics, 2019) was used to computationally predict the ADME parameters of sixty-one secondary metabolites from white cabbage, red onion, and coffee extracts. Pharmacokinetic profiles were assessed using Lipinski's “rule of five,” which examines a drug's biochemical characteristics that may affect how well it absorbs and permeates through cell membranes. A compound must meet at least three of Lipinski's criteria (molecular weight <500 Da, estimated lipophilicity (log P) <5, number of hydrogen-bond donors <5, and number of hydrogen-bond acceptors <10) in order to show drug similarity. Additionally, for *in silico* toxicity prediction, which considers the possible mutagenicity, tumorigenicity, irritating effects, and reproductive toxicity of the secondary metabolites from white cabbage, red onion, and coffee extracts, the OSIRIS property explorer program (Thomas Sander, Idorsia Pharmaceuticals Ltd., 2017) was utilized [20].

### 2.7. *In Vitro* PDE4B1 Inhibitory Assay

PDE4B1 inhibitory assay was performed in accordance with the manufacturer's protocol using certain concentrations of white cabbage extract (100, 50, 25, 12.5, 6.25, 3.125, 1.5625 *µ*g/mL), coffee extract (100, 50, 25, 12.5, 6.25, 3.125, 1.5625 *µ*g/mL), and red onion extract (100, 50, 25, 12.5, 6.25, 3.125, 1.5625 *µ*g/mL) and the combination (white cabbage extract (100, 50, 25, 12.5, 6.25, 3.125, 1.5625 *µ*g/mL), coffee extract (100, 50, 25, 12.5, 6.25, 3.125, 1.5625 *µ*g/mL), and red onion extract (100, 50, 25, 12.5, 6.25, 3.125, 1.5625 *µ*g/mL)) extracts. The same concentrations were applied for sinigrin, chlorogenic acid, and quercetin. In this protocol, the fluorescently labeled cAMP was incubated with a sample containing PDE4B1 for 1 hour, and a binding agent which was added to the reaction mixture to induce changes in fluorescent polarization was then measured using a fluorescence reader equipped for the measurement of fluorescence polarization, which was measured at 485 ± 5 nm (excitation) and 528 ± 10 nm (emission) using a properly equipped microtiter-plate fluorescence reader.

### 2.8. Statistical Analysis

The data are presented as the mean ± standard errors of means (SEM). To generate sigmoidal % activity versus concentration graphs, IC_50_ was calculated using nonlinear regression curve fitting. One-way ANOVA was applied for analysis followed by Tukey's multiple comparison test, and a *p* value of 0.05 was established as the statistically significant value. GraphPad Prism version 5.0 was used to analyze the data.

## 3. Results

### 3.1. Identification of Chief Bioactive Compounds by TLC-densitometry Analysis

**Figure 1:**
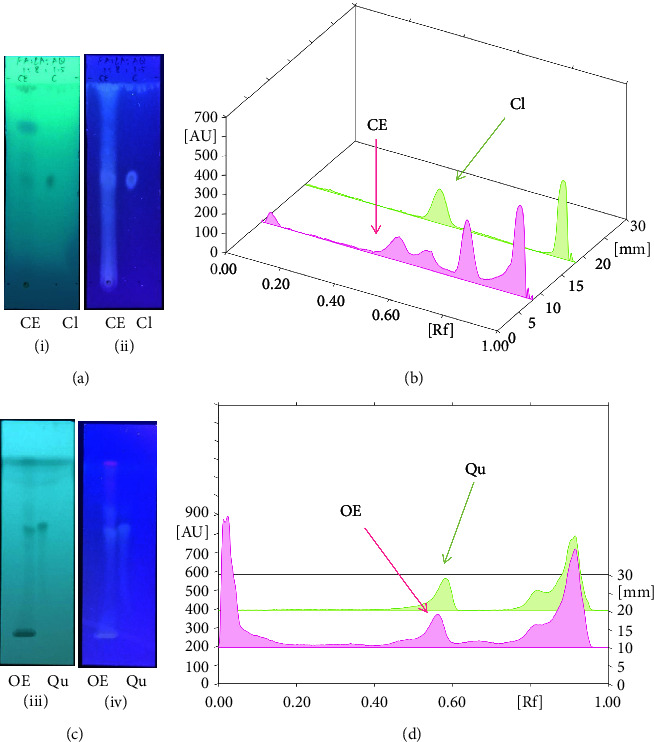
TLC profile of coffee, onion, chlorogenic acid, and quercetin along with their spectrums (b) and (d). TLC plates indicate (a(i)) and (c(iii)) (at 254 nm); (a(ii)) and (c(iv)) (at 366 nm); CE = coffee extract; OE = onion extract; Cl = chlorogenic acid; Qu = quercetin; AUC = area under the curve; Rf = retention factor.

We identified chlorogenic acid in coffee. On a silica-gel F254 plate, the compound was detected at UV 254 and 366 nm (Figures 1(a)(i) and 1(a)(ii), respectively). TLC-densitometry analysis was performed to confirm this finding (Figure 1(b)). Quercetin was detected at UV 254 and 366 nm in red onion (Figures 1(c)(iii) and 1(c)(iv), respectively). This result was confirmed by TLC-densitometry analysis (Figure 1(c)).

### 3.2. *In Silico* Molecular Docking Studies

**Figure 2:**
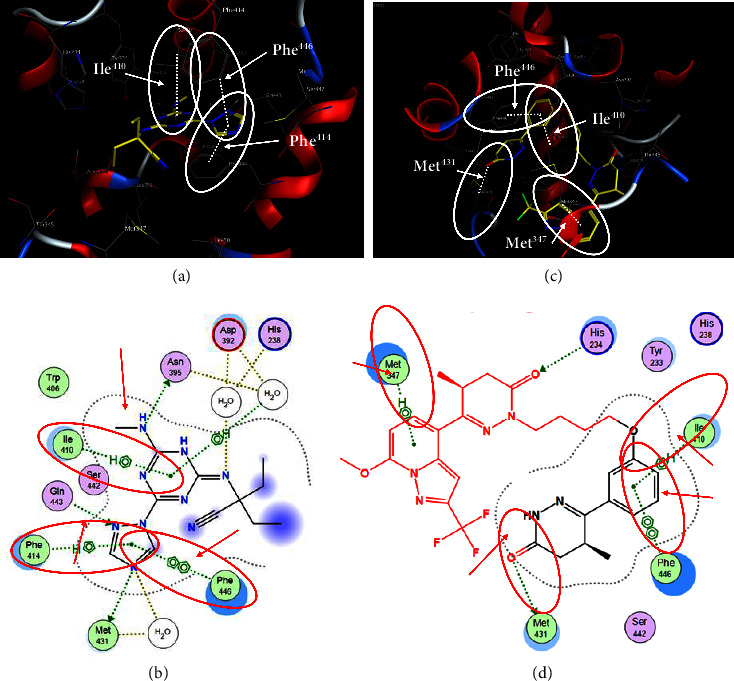
The 3D caption of native and known ligands with native ligand, and the 2D caption of native ligand and known ligands with amino acid residues. (a) = redocked native ligand 3D interaction with amino acid residues; (b) = native ligand 2D interaction with amino acid residues; (c) = docked known ligand (ZINC000043194322) 3D interaction with amino acid residues; (d) = known ligand (ZINC000043194322) 2D interaction with amino acid residues. Trp = tryptophan; Phe = phenylalanine; Asp = aspartic acid; Gln = glutamine; Met = methionine; Ser = serine; Ile = isoleucine; His = histidine; H = hydrogen; N = nitrogen; F = fluorine; O = oxygen; H_2_O = water.

Figure 2(a) exhibits captioned 3D images of the interaction between a redocked native ligand [(2-ethyl-2-{[4-(methylamino)-6-(1H-1,2,4-triazol-1-yl)-1,3,5-triazin-2-yl]amino}butanenitrile) (PubChem CID: 71598545)] and amino acids of PDE4B (PDB ID: 4KP6) with an RMSD value of 0.834 Å and binding energy (−21.494 kcal/mol). Figure 2(b) shows their 2D interaction. In the current docking research, it was discovered that the triazine ring of native ligand had a *π*-hydrogen bonding with Ile410 and its nitrogen on amine group on the same ring with Asn395 while its triazole ring had *π*-hydrogen interaction with Phe414 and *π*–*π* binding with Phe446; besides this, nitrogen in its triazole ring represented attachment with Met431 (Figures 2(a) and 2(b)). On the other hand, the known ligand (ZINC000043194322) (binding energy −35.123 kcal/mol) was found to interact in 2D with amino acids of PDE4B (PDB ID: 4KP6) in Figure 2(c), whereas Figure 2(d) represents a known ligand's 2D association with amino acids. Additionally, the known ligand's benzene ring displayed a *π*–*π* interaction with Phe446, and the pyridine ring had *π*-hydrogen bonding with Met347; however, the oxygen on the pyrimidine ring served as a sidechain donor at Met431 (Figures 2(c) and 2(d)).

**Figure 3:**
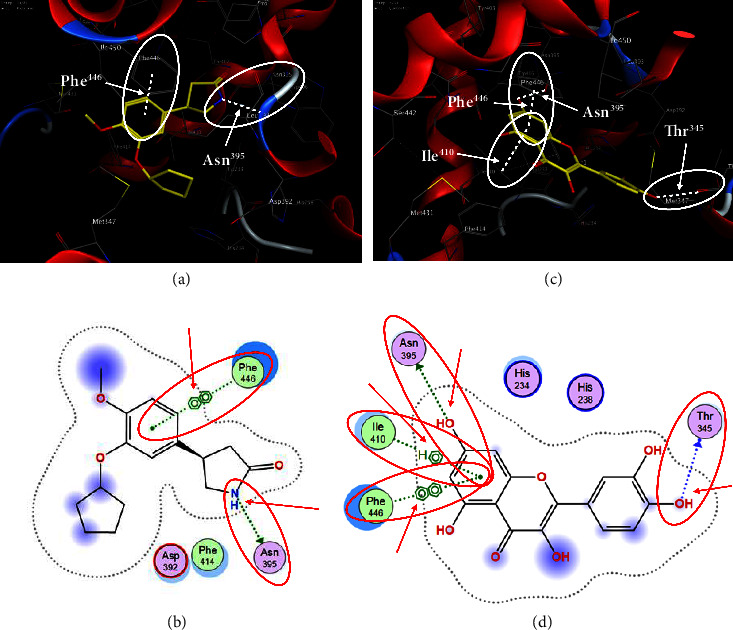
The 3D caption of rolipram and quercetin with amino acid residues, and the 2D caption of rolipram and quercetin with amino acid residues. (a) = rolipram 3D interaction with amino acid residues; (b) = rolipram 2D interaction with amino acid residues; (c) = quercetin 3D interaction with amino acid residues; (d) = quercetin 2D interaction with amino acid residues. Phe = phenylalanine; Asp = aspartic acid; Thr = threonine; Ile = isoleucine; His = histidine; N = nitrogen; H = hydrogen; O = oxygen; OH = hydroxyl group.

Quercetin (PubChem CID: 5280343), the bioactive component in red onion, and reference drug rolipram (PubChem CID: 5092) have also been studied to find out how well they interact with PDE4B (PDB ID: 4KP6).  Figure 3 shows the 3D interaction of rolipram (binding energy −22.801 kcal/mol) with amino acids of PDE4B (PDB ID: 4KP6) in Figure 3(a) and the 2D interaction in Figure 3(b). This study revealed that the nitrogen in the pyrrolidine ring in rolipram contributed sidechain to Asp^395^ and the benzene ring interacted with Phe^446^ by *π*–*π* binding interaction (Figures 3(a) and 3(b)). Alternatively, Figure 3(c) exhibits 3D interaction of quercetin (binding energy −17.252 kcal/mol) with amino acids of PDE4B (PDB ID: 4KP6) while Figure 3(d) represents 2D interaction of known ligand with amino acids. The quercetin's benzene-1,2-diol ring interacted with Phe^446^ through *π*–*π* binding and *π*-hydrogen binding with Ile^410^, while its hydroxyl group served as a donor to Asn^395^ in these interactions. Moreover, the hydroxyl group on the benzene-1,2-diol ring of quercetin served as a donor to Thr^414^ (Figures 3(c) and 3(d)).

**Figure 4:**
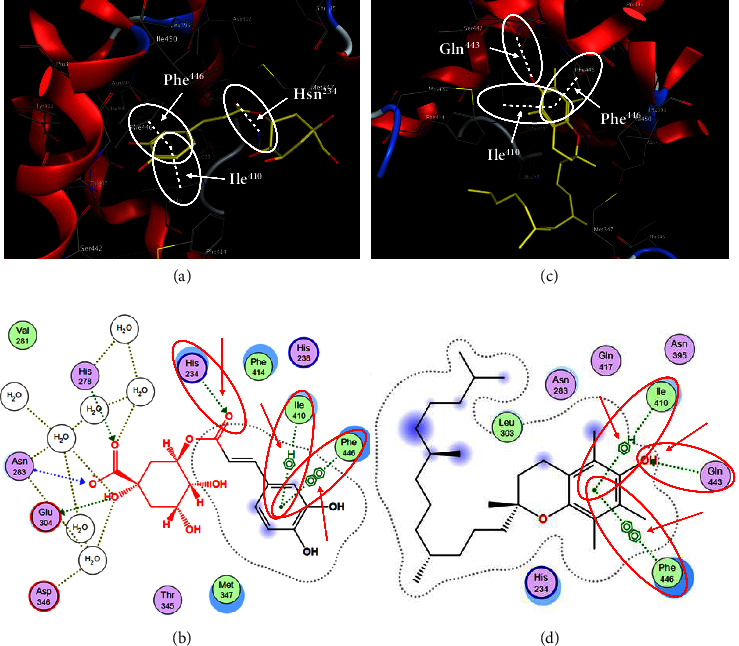
The 3D caption of chlorogenic acid and alpha-tocopherol with native ligand, and the 2D caption of chlorogenic acid and alpha-tocopherol with amino acid residues. (a) = chlorogenic acid 3D interaction with amino acid residues; (b) = chlorogenic acid 2D interaction with amino acid residues; (c) = alpha-tocopherol 3D interaction with amino acid residues; (d) = alpha-tocopherol (PubChem CID 1742129) 2D interaction with amino acid residues. Phe = phenylalanine; Asn = asparagine; Glu = glutamic acid; Asp = aspartic acid; Gln = glutamine; Met = methionine; Thr = threonine; Ile = isoleucine; Val = valine; His = histidine; H = hydrogen; O = oxygen; OH = hydroxyl group; H_2_O = water.

In order to evaluate chlorogenic acid (PubChem CID: 1794427) and alpha-tocopherol's (PubChem CID: 1742129) affinity for the PDE4B (PDB ID: 4KP6), chlorogenic acid, a coffee bioactive component, has also been studied. In Figure 4, Figure 4(a) depicts the 3D interaction of chlorogenic acid (binding energy −21.501 kcal/mol) with amino acids of PDE4B (PDB ID: 4KP6), while Figure 4(b) shows the 2D interaction. This finding exhibited that the benzene ring in benzene-1,2-diol of chlorogenic acid interacted with Phe^446^ by *π*–*π* binding and Ile^410^ by *π*-hydrogen binding, and oxygen on carboxylic acid in chlorogenic acid served as side chain acceptor for His^234^ while hydroxyl group acted as side chain donor interaction of the known ligand with amino acids. Additionally, the alpha-tocopherol's indole ring showed an engagement with Phe^446^ through *π*–*π* binding and Ile^410^ by *π*-hydrogen binding while the hydroxyl group on the benzene ring served as a side chain acceptor for Gln^443^ (Figures 4(c) and 4(d)). The active component of white cabbage, sinigrin, has also been investigated to determine its affinity for PDE4B (PDB ID: 4KP6). Unfortunately, sinigrin from white cabbage extract did not interact with PDE4B in all scoring functions. In terms of binding interactions with functional residues, the docking results of alpha-tocopherol were investigated excellently, and it also had the strongest effects on the PDE4B (PDB ID: 4KP6) target protein out of the 28 phytochemicals, including rolipram, that were chosen for Glu^304^ (Figures 4(a) and 4(b)). In contrast, Figure 4(c) represents the 3D interaction of alpha-tocopherol (binding energy = −38.007 kcal/mol) with amino acids of PDE4B (PDB ID: 4KP6), whereas Figure 4(d) exhibits 2D.

In the table (Table 2), the compounds' names, their structures (along with rolipram, as a reference), free bind energies (∆G_bind_ [kcal/mol {kilocalorie/molar}]), and pIC_50_ values have been enlisted.

### 3.3. ADME and Drug-Likeness Prediction

**Figure 5:**
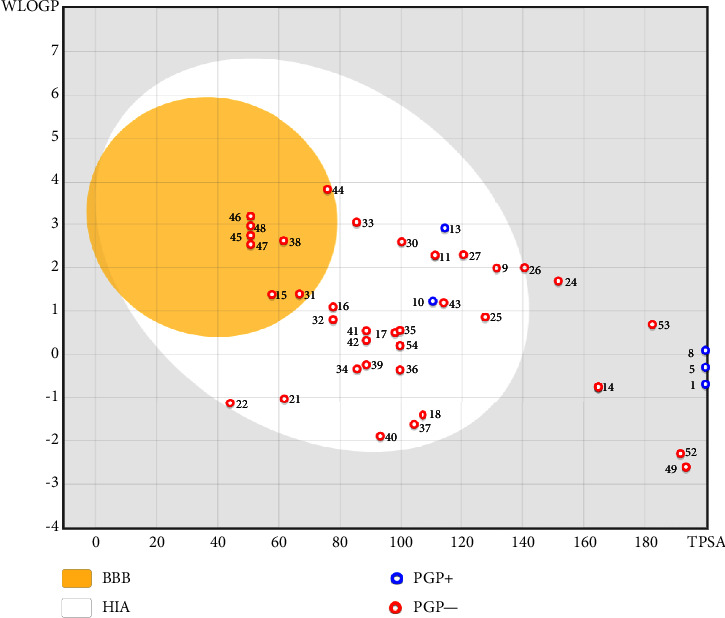
GI tract and brain permeation prediction of the 41 top-ranked secondary metabolites in white cabbage, red onion, and coffee by brain or the intestinal estimated permeation predictive model (BOILED-Egg) method. GI = gastrointestinal, BBB = blood-brain barrier, HIA = human intestinal absorption, PGP+ = P-glycoprotein positive, PGP- = P-glycoprotein negative, TPSA = topological polar surface area, secondary metabolites: 1 = sinigrin, 5 = gluconapin, 8 = glucobrassicanapin, 9 = quercetin, 10 = catechin, 11 = kaempferol, 13 = cyanidin, 14 = chlorogenic Acid, 15 = p-coumaric acid, 16 = caffeic acid, 17 = gallic acid, 18 = ascorbic acid, 21 = caffeine, 22 = trigonelline, 24 = myricetin, 25 = taxifolin, 26 = laricitrin, 27 = isorhamnetin, 30 = tectorigenin, 31 = ferulic acid, 32 = protocatechuic acid, 33 = onionin (A) 34 = cycloalliin, 35 = isoalliin, 36 = methiin, 37 = propiin, 38 = allicin, 39 = S-methyl-L-cysteine, 40 = S-ethylcysteine, 41 = S-propyl-L-cysteine, 42 = S-allylcysteine, 43 = S-propylmercaptocysteine, 44 = dipropyl trisulfide, 45 = diallyl disulfide, 46 = diisopropyl disulfide, 47 = methyl propenyl disulfide, 48 = 6-methyl-4,5-dithia-1-heptene, 49 = cyanidin 3-glucoside, 52 = malvidin 3-glucoside, 53 = peonidin-3-glucoside, and 54 = alliin.

Through the use of *in silico* absorption, distribution, metabolism, and excretion (ADME) screening, the overall pharmacokinetic behavior of 61 compounds was defined. Using the following descriptors—molecular weight, lipophilicity (given as octanol-water partition coefficient), and the number of hydrogen-bond donors and acceptors—the druggability of the compounds was predicted using Lipinski's rule of five. Partial bioavailability and drug-likeness were demonstrated by all secondary metabolites, which included forty-one highly ranked compounds. Furthermore, passive intestinal absorption and brain permeation of the substances were predicted using the intuitive graphical representation of the functions of lipophilicity and apparent polarity known as the BOILED-Egg (brain or intestinal estimated permeation predictive model) (Figure 5).

The BBB penetration prediction is very probable for the eight compounds (p-coumaric acid, ferulic acid, allicin, dipropyl trisulfide, diallyl disulfide, diisopropyl disulfide, methyl propenyl disulfide, and 6-methyl-4,5-dithia-1-heptene) present in the yellow region (yolk), but passive absorption through the gastrointestinal (GI) tract is more likely for those in the white region. With the significant binding affinity to the target enzyme PDE4B, the twenty-five compounds (quercetin, catechin, kaempferol, cyanidin, caffeic acid, gallic acid, ascorbic acid, caffeine, trigonelline, taxifolin, laricitrin, isorhamnetin, tectorigenin, protocatechuic acid, onionin A, cycloalliin, isoalliin, methiin, propiin, S-methyl-L-cysteine, S-ethylcysteine, S-propyl-L-cysteine, S-allylcysteine, S-propylmercaptocysteine, and alliin) were expected to have low BBB bridging capacities and good GI absorption. The bioavailability pattern of diallyl disulfide, diisopropyl disulfide, methyl propenyl disulfide, and 6-methyl-4,5-dithia-1-heptene was similar. Sinigrin, gluconapin, and glucobrassicanapin's location outside of the white region of the BOILED-Egg model can be explained by its expected poor absorption in the GI tract, even though it has a good binding affinity to PDE4B. The OSIRIS Property Explorer was also utilized to estimate the reproductive toxicity, mutagenicity, and tumorigenicity, irritating impact toxicities of the sixty-one secondary metabolites derived from white cabbage, red onion, and coffee extracts. The thirty-four compounds (catechin, luteolin, chlorogenic acid, ascorbic acid, cryptochlorogenic acid, neochlorogenic acid, trigonelline, taxifolin, hyperoside, onionin A, cycloalliin, isoalliin, methiin, propiin, allicin, S-methyl-L-cysteine, S-ethylcysteine, S-propyl-L-cysteine, S-allylcysteine, S-propylmercaptocysteine, dipropyl trisulfide, diallyl disulfide, diisopropyl disulfide, methyl propenyl disulfide, 6-methyl-4,5-dithia-1-heptene, cyanidin 3-glucoside, malvidin 3-glucoside, alliin, rutin, violaxanthin, alpha-tocopherol, neoxanthin, quercetin 3,4′-diglucoside, and *β*-carotene) did not show any predicted toxicity among the top-ranked compounds. However, the rest twenty-three compounds exhibited potential toxicities, including mutagenicity, tumorigenic, irritant, and reproductive effective toxicities. It was predicted that sinigrin, progoitrin, 4-methoxyglucobrassicin, glucobrassicin, 4-hydroxyglucobrassicin, glucoalyssin, chlorogenic acid, p-coumaric acid, and tectorigenin would be harmful to reproduction while gluconapin and glucobrassicanapin were expected to have irritant and reproductive effects. Caffeic acid, caffeine, and ferulic acid would be predicted as highly dangerous due to possessing mutagenicity, tumorigenic, and reproductive effective properties. Moreover, kaempferol, quercetin-4′-o-glucoside, myricetin, laricitrin, isorhamnetin, spiraeoside, protocatechuic acid, and quercetin 3,4′-diglucoside expected to have mutagenicity effects (Table 3). Therefore, to enhance their toxicity profile, structural iteration is strongly advocated.

### 3.4. *In Vitro* PDE4B1 Assay

**Figure 6:**
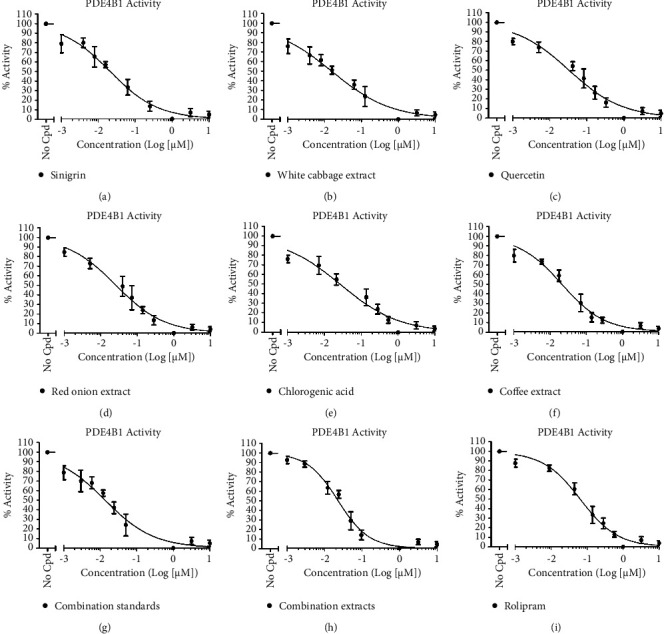
Nonlinear regression curves between the activity (%) of nine tested samples against PDE4B1 and their concentrations (Log (*µ*M)). (a) = sinigrin (IC50 = 0.24 ± 0.01 *µ*M); (b) = white cabbage extract (IC50 = 0.27 ± 0.009 *µ*M); (c) = quercetin (IC50 = 0.25 ± 0.01 *µ*M); (d) = red onion extract (IC50 = 0.22 ± 0.02 *µ*M); (e) = chlorogenic acid (IC50 = 0.20 ± 0.03 *µ*M); (f)=coffee extract (IC50 = 0.18 ± 0.03 *µ*M); (g) = combination (sinigrin, quercetin, and chlorogenic acid) standard (IC50 = 0.17 ± 0.03 *µ*M); (h) = combination (white cabbage, red onion, and coffee) extract (IC50 = 0.12 ± 0.03 *µ*M); (i) = rolipram (a PDE4B inhibitor) (IC50 = 0.15 ± 0.008 *µ*M). PDE4B1 = phosphodiestrase-4B1; No Cpd = no compound.

An *in vitro* assay was performed to confirm the activities of the tested extracts, combination extract (white cabbage, red onion, and coffee), standards, and combination standard (sinigrin, quercetin, and chlorogenic acid) (Figure 6; Table 4). All samples were assayed at a concentration of 100 *µ*g/ml with serial dilutions, and it was found that the sinigrin standard showed a very weak PDE4B1 inhibitory activity (Figure 6(a); Table 4); alternatively, white cabbage extract (Figure 6(b); Table 4) presented higher PDE4B1 inhibition than sinigrin standard. Similarly, the quercetin standard (Figure 6(c); Table 4) displayed PDE4B1 inhibitory activity which was greater than white cabbage extract but less than red onion extract (Figure 6(d); Table 4). Moreover, red onion extract showed this effect significantly more than the quercetin standard, and it is probably owing to the rest compounds such as polyphenols and organosulfates present in the extract. Likewise, chlorogenic acid (Figure 6(e); Table 4) exhibited PDE4B1 inhibition more than red onion while less than coffee extract (Figure 6(f); Table 4). The coffee extract showed a PDE4B1 inhibitory effect significantly greater than chlorogenic acid, and this maybe due to additional compounds such as caffeine present in the extract. Lastly, it was observed that combination extract (Figure 6(h); Table 4) represented significantly excellent PDE4B1 inhibitory activity with IC_50_ at a concentration of 0.12 ± 0.03 *µ*M among all under test standard and reference samples, even over combination standard (Figure 6(g); Table 4); however, our results were in accordance with the reference (rolipram). In the present study, rolipram (Figure 6(i); Table 4) was employed as a reference drug against all the test extracts and their standards and exhibited a PDE4B1 inhibitory activity slightly less than combination extracts, and contrarily, near to combination standard. IC_50_ values (PDE4B1 inhibitory activities) of test compounds, extracts, and rolipram are summarized in Table 4 while Figure 6 represents nonlinear regression curves between PDE4 B1% activity and concentration (Log (*µ*M)) of nine tested samples.

## 4. Discussion

Plants are the hidden source of mysterious therapeutic components [209]. Over the last several decades, the therapeutic value of natural products has long gone unreported in health services [210]. Alternatively, numerous available data indicate that people used to consume herbs to treat a variety of ailments [211]. Contrary to popular belief, plants play a crucial role in the provenance of current allopathic medications [212]. Plant products have been examined based on folklore uses to enhance the quality of life and define the scientific horizon [213]. Cabbage, coffee, and red onion are rich sources of glucosinolates, phenolic acids, and flavonoids, respectively. These phytochemicals participate in controlling multiple diseases, such as neurodegenerative diseases, especially memory dysfunction by targeting PDE4 [30, 39, 40, 46, 47]. The chemical profile of coffee and red onion extracts was analyzed using a TLC-densitometry. Coffee and red onion extracts were dissolved in methanol as stated in the method section and analyzed on a precoated silica-gel TLC plate for the identification of chlorogenic acid and quercetin as major compounds. The current study detected chlorogenic acid and quercetin in coffee and red onion, respectively, and evaluated their *in silico* and *in vitro* activities against PDE4B1 as well as pharmacokinetic and toxicity studies. In our previous study, Ahmad et al. 2023 followed the same procedure that identified sinigrin (with an Rf 0.77 at maximum wavelength 399 nm) in white cabbage by using TLC-densitometry analysis [214]; hence, we excluded sinigrin in our current analysis.

Molecular docking was carried out to gain insight into the binding affinity and potential interaction of PDE4B (PDB ID: 4KP6) with the bioactive compounds of white cabbage, coffee, and red onion. The protein targets were validated (redocked) prior to conducting the docking research, and RMSD values were used as a parameter. RMSD is a distinctive characteristic that demonstrates the capacity of both native ligand complexes and proteins to duplicate themselves in the development of an appropriate configuration. The preferred RMSD value is 1 Å, though 2 Å is also appropriate [215]. Sixty-three compounds were tested against PDB4B (PDB ID: 4KP6) by performing their molecular docking with MOE. The authors found that 28 compounds interacted with PDE4B (PDB ID: 4KP6) by binding majorly with amino acid residues (Phe^446^ and Ile^410^); however alpha-tocopherol, which is one of the previously reported bioactive components of white cabbage, exhibited the strongest binding affinity for PDE4B (PDB ID: 4KP6) by interacting with Phe^446,^ Ile^410^, and Gln^443^ residues while chlorogenic acid with Phe^446^, Ile^410^, and His^234^ and quercetin with Phe^446,^ Ile^410^, and Asp^395^ interacted moderately. Unfortunately, sinigrin did not represent any interaction with PDE4B (PDB ID: 4KP6). The present study also exhibits that rolipram showed its affinity by creating an attachment with Phe^446^ and Asp^392^ residues. In an earlier investigation, test ligands interacted with PDE4 (1XMU) amino acids (Phe^446^, Asn^395^, Gln^443^, and Ile^410^) [216]. Similarly, in another study, rolipram generated a contact with Phe^446^, Asp^392^, and Ile^410^ residues of PDE4 [217]. It means that interaction with any one of the amino acids, such as Phe^446^, Asp^392^, and Ile^410^, can result in the inhibition of PDE4B activity. Based on these investigations, our docking results are in accordance with Xia et al. reports [216, 217]. ∆*G*_bind_ is a crucial parameter because it shows how strongly compounds attach to the receptor. Even, the stability and strength of the interaction between an enzyme and its ligand are also indicated by a meager ∆*G*_bind_. The pharmacological impacts are influenced by ∆*G*_bind_ [215]. The compounds' ∆*G*_bind_ was identical to one another, with the standard compound having the lowest free-bond energy. As a result, white cabbage, coffee, and red onion can be able to target PDE4B because their compounds (sinigrin, chlorogenic acid, and quercetin, respectively) interacted with PDE4B during docking, and they may also be an excellent source of a natural cognitive enhancer that can help treat memory impairment. Therefore, it can be suggested that white cabbage, coffee, and red onion extracts should be researched more thoroughly in *in vivo* investigations.

Drug development has advanced to a new stage that uses computer-based drug design in predicting the ADME characteristics of the medications [218]. A free online tool called SwissADME was used to assess the pharmacokinetics, drug-likeness, and medicinal chemistry friendliness of small compounds. Most of the compounds among sixty-one compounds from white cabbage, red onion, and coffee extracts exhibited to have no predictive toxicities during computational analysis. However, one by third part of the analyzed compounds possess predictive harmful effects which were predicted by SwissADME and OSIRIS. In a previous study, though there was a low GI absorption, quinadoline pharmacophore had an adequate ADME profile with logP and solubility in acceptable limits [219]. The findings from brain and intestinal estimated permeation predictive model (BOILED-Egg) approach for the GI tract and brain permeation for the prediction of the top-ranked secondary metabolites were corroborated with the recent earlier study [20]. The results of this study indicate that additional research on the bioactive compounds of white cabbage, red onion, and coffee extracts against their respective target proteins is necessary to find new therapeutic leads against memory function and AD, as the top-ranked ligands have good pharmacokinetic features.

The idea of virtual screening of secondary metabolites from white cabbage, coffee, and red onion extracts was sparked by the high global prevalence of memory impairments and the absence of effective and safe treatments. According to the previous studies, the computational identification of kobophenol A as a promising lead drug against SARS-CoV-2 infection was confirmed through experimental validation, demonstrating its ability to prevent the binding of S1-RBD from SARS-CoV-2 to the host ACE2 receptor. The data obtained indicated that kobophenol A might be explored further as a pharmacological treatment for SARS-CoV-2 infection that is safe, efficacious, and nontoxic [220]. According to the *in silico* ADMET prediction, quinadoline B confers high drug-likeness, poor blood-brain barrier penetrability, and high gastrointestinal absorption, in accordance with Lipinski's rule of five. The complexes generated between the top-ranked ligands and their respective protein targets were dynamically stable and had high total free energy of binding, as demonstrated by MD modeling and subsequent total free energy calculation. Consequently, these substances may serve as models or models for the creation of multitargeting ligands directed against SARS-CoV-2 [221]. In another study, MD simulations were performed on the compounds that rated highest in order to assess the stability of the systems over a predetermined period of time and to thoroughly examine the binding mechanism of the most promising derivatives. Ultimately, five compounds with a better affinity for SARS-CoV-2 RdRp were found using the *in silico* searching procedure, piqueing researchers' interest in pursuing additional research on these compounds as potential antiviral medicines. Notably, the suggested computational methodology offers a convenient computational procedure for hit-to-lead optimization, with implications for anti-SARS-CoV-2 drug development and generally in the drug optimization process [219]. One more study revealed that the complexes formed between the protein targets of the top-ranked ligands were found to be thermodynamically stable by MD modeling. These results, along with the advantageous pharmacokinetic characteristics of the top-ranked ligands, call for additional research on *U. alba*'s secondary metabolites in relation to their individual target proteins in order to identify novel therapeutic approaches against AD and cancer [20]. However, the current work on cabbage, coffee, and red onion extracts could serve as a foundation for the future search and creation of unique cognitive-enhancing drugs.

PDE4B is widely expressed in the different brain regions. PDE4B1, a long isoform of PDE4B [222], is mainly present in hippocampal CA2 and CA3 regions being more specific than the PDE4B gene knockouts, thereby considering it a possible therapeutic target in cognition by promoting neuroplasticity [222, 223] while PDE4B2B, a short isoform, usually expressed in cortex and hippocampus [224] and imparts crucial role in neuroinflammation [225, 226]. Its structural superposition showed a little conformational change, suggesting that the inhibitors might not be PDE4 subfamily-specific [227]. To determine the IC_50_ values of the tested compounds (sinigrin, quercetin, and chlorogenic acid and their combination), white cabbage, coffee, and red onion and their combination extracts against PDE4B1, an *in vitro* assay was performed. Rolipram was used as a reference drug for PDE4B1 inhibitory activity. This study revealed different IC_50_ values between sinigrin and white cabbage extract against PDE4B1 that may be due to the extract containing other phytoconstituents. In one study, sinigrin enhanced intracellular cAMP levels by PDE4 inhibition at its highest concentration 10^−5^ mol/L [30]. However, literature is still lacking studies on sinigrin and white cabbage extract in connection with PDE4B1. Likewise, quercetin and red onion extract were assayed against PDE4B1 in order to determine IC_50_ values. Our findings indicated that red onion extract had more PDE4B1 inhibitory activity as compared to quercetin, which is a naturally occurring PDE4-selective inhibitor present in vegetables, tea, and fruits [228]. Although quercetin inhibitory activity for PDE4D is available currently, a study gap for quercetin and red onion extract on PDE4B1 is still present. However, only quercetin 3-*O–α-L*-arabinopyranosyl-(1⟶2)-*O–α-L*-rhamnopyranoside, a derivative of quercetin, was found to show inhibitory activity against PDE4B with an inhibition 100% in an *in vitro* assay [229]. Similarly, chlorogenic acid and coffee extract were assayed against PDE4B1 in order to examine their PDE4B1 inhibitory activities. The results of the current study indicated that coffee extract had more PDE4B1 inhibitory activity as compared to chlorogenic acid. Though chlorogenic acid was evaluated against PDE4 only in previous studies, the data are not available on chlorogenic acid and coffee extract against PDE4B1. The authors employed rolipram as a reference drug against tested compounds (sinigrin, quercetin, and chlorogenic acid and their combination), combination (white cabbage, coffee, and red onion and their combination) extracts for PDE4B1 inhibitory activity. Our investigation exhibited that rolipram inhibited PDE4B1 more than all tested samples except combination extracts. In a previous study, Xia et al. determined the PDE4B1 inhibitory activity of rolipram in an *in vitro* fluorescence polarization assay where it showed IC_50_ at a concentration of 0.13 ± 0.02 *µ*M [216]. Additionally, rolipram was also analyzed against PDE4B1 in an *in vitro* enzymatic assay and showed IC_50_ at a concentration of 0.15 ± 0.02 *µ*M by Tang et al. [230]. Our results for all test compounds, extracts, and reference rolipram were consistent with the earlier studies against PDE4 B. As per the previous report, a single-dose triple combination has more efficacy, safety, and fewer side effects than high-dose single therapy and/or twice-a-day double therapy [231]. Therefore, based on our *in vitro* experiment, a single-dose triple combination of extracts treatment can be safer and more effective than mono and double extracts in treating memory function. However, a further, *in vivo* approach is guaranteed to strengthen this evidence.

**Figure 7:**
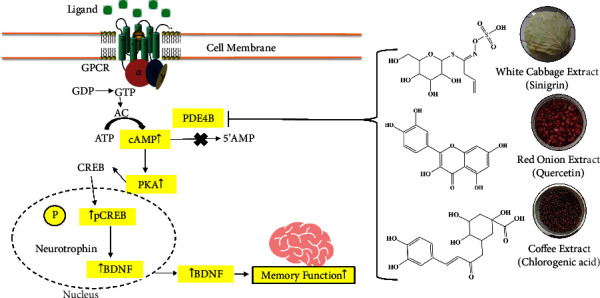
Mechanism of action of cabbage, coffee, and red onion extracts. GPCR = G-protein coupled receptor, GDP = guanosine diphosphate, GTP = guanosine triphosphate, AC = adenylyl cyclase, ATP = adenosine triphosphate, cAMP = cyclic adenosine monophosphate, 5′-AMP = 5-adenosine monophosphate, PKA = protein kinase (A) BDNF = brain-derived neurotrophic factor, CREB = cAMP-response element binding, pCREB = phosphorylated cAMP-response element binding.

To explain how cabbage, coffee, and red onion extracts work, the author made a proposed mechanism of action targeting PDE4B that can help in further *in vivo* studies and drug development as well (Figure 7). According to the current findings of the molecular analysis, cabbage, coffee, and red onion extracts work to prevent memory loss by acting as a PDE4B inhibitor. The previous study embarks that inhibition of PDE4B enzyme triggers the activation of cAMP/PKA/CREB/BDNF signaling pathway and subsequently raises the expression of BDNF protein in the hippocampus, thereby improving memory function [232]. According to the previously reported study findings [30, 40, 46], chlorogenic acid, quercetin, and sinigrin from coffee, red onion, and white cabbage extracts, respectively, have both *in vitro* and *in vivo* PDE4 inhibitory actions. Effective methods for raising intracellular cAMP levels include inhibition of this enzyme which is beneficial for improving memory function [30, 39, 46, 233]. The cabbage, coffee, and red onion extracts activate the adenylate cyclase pathway, as cAMP/PKA/CREB/BDNF pathway, thereby increasing the level of BDNF, a protein found in synapses, BDNF controls the expansion, upkeep, and maturation of brain neurons, expression in the hippocampus and preventing the memory loss. The brain's temporal lobe, cerebral cortex, and hippocampal regions are where this protein is primarily expressed. BDNF is crucial for preserving the form and functionality of neurons. A deficiency of neurotrophic protein can raise the risk of neuronal loss, which can cause damage and death to brain neurons. This loss of neurons impairs brain function physiologically and results in cognitive issues, including memory loss [1]. However, the compounds present in white cabbage, coffee, and red onion extracts have the ability to block PDE4B and reverse memory loss.

It is unknown if PDE4B negatively influences the pathway involved in CREB-mediated neuronal survival. The discovery of PDE4B-selective inhibitors has opened up new avenues for research into the dual function of PDE4B as a pro-survival and anti-inflammatory target for a variety of neurological conditions and traumas especially in cognition.

## 5. Conclusion

In summary, quercetin and chlorogenic acid were identified by TLC-densitometry as the major compounds in red onion and coffee, respectively. The molecular docking study showed that among 28 bioactive components, alpha-tocopherol had the best binding interaction with the PDE4B whereas chlorogenic acid and quercetin demonstrated moderate interaction. In this experiment, sinigrin did not show the interaction with PDE4B. The majority of compounds presented no toxicities. The combination extracts showed stronger inhibition against PDE4B1 as compared to the combination standard and reference (rolipram). Further research on an *in vivo* model is needed to confirm this activity.

## Figures and Tables

**Table 1:** List of 67 reported test compounds from white cabbage, coffee, and red onion extracts excluding six repeated components.

Sr. no.	Test compound	Structure	Biological activity	References
1. White cabbage				
A	Glucosinolates			

1	Sinigrin (PubChem ID: 6911854)	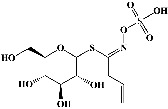	Wound healing and antiproliferative properties	[52–54]
2	Progoitrin (PubChem ID: 5281139)	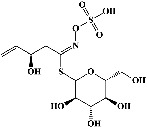	Antithyroid activity	[52, 55]
3	4-Methoxyglucobrassicin (PubChem ID: 9576738)	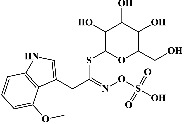	Antioxidative activity	[52, 56]
4	Glucobrassicin (PubChem ID: 6602378)	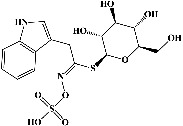	Antioxidant activity	[52, 57]
5	Gluconapin (PubChem ID: 9548620)	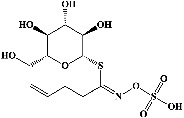	Hypotriglyceridemia	[52, 58]
6	4-Hydroxyglucobrassicin (PubChem ID: 656561)	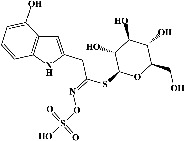	Antioxidant activity	[52, 59]
7	Glucoalyssin (PubChem ID: 656523)	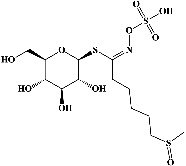	Antimicrobial activity	[60, 61]
8	Glucobrassicanapin (PubChem ID: 5485207)	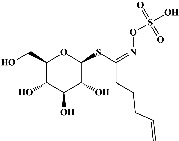	Antioxidant activity	[60, 62]

B	Flavonoids			

9	Rutin (PubChem ID: 5280805)	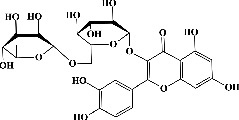	Anticancer, antioxidant, anti-inflammatory, and antiamyloidogenic activities	[63–66]
10	Quercetin (PubChem ID: 5280343)	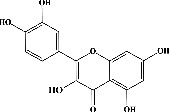	Antiamyloidogenic, antioxidant, and anti-inflammatory activities	[63, 66, 67]
11	Catechin (PubChem ID: 9064)	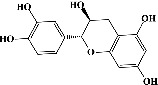	Antioxidant, antibacterial, and antimetastatic activities	[63, 68–70]
12	Kaempferol (PubChem ID: 5280863)	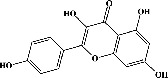	Antimicrobial, antioxidant antitumor, anti-inflammatory and antiproliferative activities	[63, 71–73]
13	Luteolin (PubChem ID: 5280445)	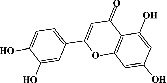	Antiviral, anti-inflammatory, antioxidant, antiallergic, and neuropharmacological activities	[63, 74–77]
14	Cyanidin (PubChem ID: 128861)	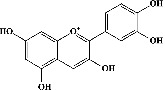	Antioxidative, anti-inflammatory, and DNA cleavage-protecting activities	[63, 78–81]

C	Phenolic acids			

15	Chlorogenic acid (PubChem ID: 1794427)	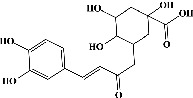	Antiulcerogenic, antioxidant, antihepatitis B virus, and DNA-protective activities	[82–87]
16	p-Coumaric acid (PubChem ID: 637542)	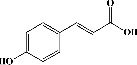	Antioxidant, antiangiogenic, and antibacterial activities	[63, 88–90]
17	Caffeic acid (PubChem ID: 689043)	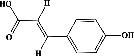	Antihepatitis B virus and antioxidant activities	[63, 85, 91, 92]
18	Gallic acid (PubChem ID: 370)	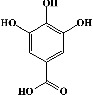	Antifungal, cytotoxicity, and antioxidant activities and antibacterial activity	[63, 93–95]

D	Other classes of compounds			

19	Alpha-tocopherol (PubChem ID: 14985)	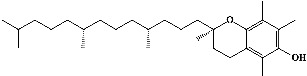	Antioxidant	[96, 97]
20	Ascorbic acid (PubChem ID: 54670067)	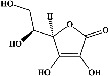	Antioxidative and anti-inflammatory activities	[98, 99]
21	*β*-carotene (PubChem ID: 5280489)	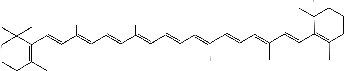	Antioxidant, antitumor, and anticancer activities	[100–103]
22	Violaxanthin (PubChem ID: 448438)	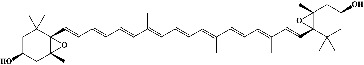	Antiproliferative, antioxidant, and anti-inflammatory	[104–108]
23	Neoxanthin (PubChem ID: 5282217)	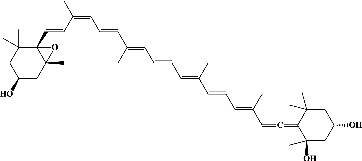	Antiproliferative and apoptosis in PC-3 human prostate cancer cells activities	[104, 109, 110]

2. Coffee				
E	Phenolic acids			

24	Chlorogenic acid^*∗*^ (PubChem ID: 1794427)	Same as no. 15	Same as no. 15	[37]
25	Cryptochlorogenic acid (PubChem ID: 9798666)	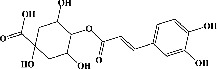	Anti-inflammatory	[111–114]
26	Neochlorogenic acid (PubChem ID: 5280633)	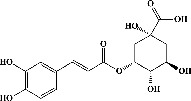	Anti-inflammatory	[111, 115]

F	Alkaloids			

27	Caffeine (PubChem ID: 2519)	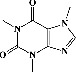	Cognitive enhancing, hepatoprotective activity	[37, 116–118]
28	Trigonelline (PubChem ID: 5570)	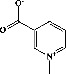	Anti-invasive, antitumor, antidiabetic, and antimicrobial activities	[37, 119–122]

3. Red onion				
G	Flavonoids			

29	Quercetin^*∗*^ (PubChem ID: 5280343)	Same as no. 10	Same as no. 10	[123]
30	Quercetin 3,4′-diglucoside (PubChem ID: 5320835)	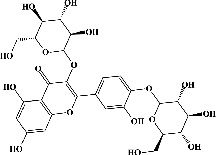	Antioxidant and anticarcinogenic activities	[124, 125]
31	Quercetin 4′-O-glucoside (PubChem ID: 12442954)	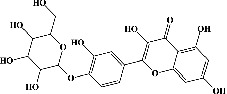	Antioxidant activity	[124, 126]
32	Myricetin (PubChem ID: 5281672)	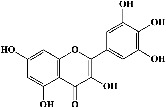	Anticancer, hypoglycemic, and anti-inflammatory activities	[127–131]
33	Taxifolin (PubChem ID: 439533)	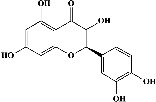	Anti-inflammatory, digestive enzymes inhibitory, antihyperglycemic, and antiglycation activities	[132–136]
34	Laricitrin (PubChem ID: 5282154)	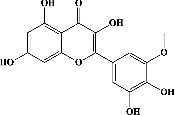	Anticancer, antioxidant, and anti-hypertension	[132, 137–139]
35	Rutin^*∗*^ (PubChem ID: 5280805)	Same as no. 9	Same as no. 9	[140]
36	Isorhamnetin (PubChem ID: 5281654)	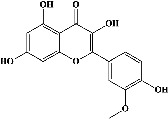	Antituberculosis, antiplatelet, antitumor, anti-inflammatory, anticancer, and antioxidant activities	[141–147]
37	Hyperoside (PubChem ID: 5281643)	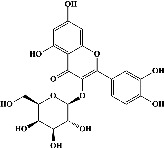	Antiviral, antifungal, antiplatelet, anticancer, and antioxidant activities	[132, 148–152]
38	Spiraeoside (PubChem ID: 5320844)	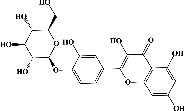	Antitumor, antioxidant, antiviral, antiradical, and enzyme inhibitory activities	[132, 153, 154]
39	Tectorigenin (PubChem ID: 5281811)	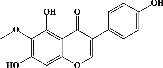	Anti-inflammatory, antioxidant, antifungal activity, and antineuroinflammatory activities	[132, 155–158]

H	Phenolic acids			

40	Ferulic acid (PubChem ID: 445858)	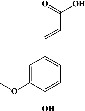	Antibacterial, antioxidant, and antimicrobial activities	[93, 159–161]
41	Protocatechuic acid (PubChem ID: 72)	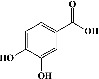	Antioxidant, antihyperlipidaemic, and antibacterial activities	[159, 162–165]
42	p-Coumaric acid^*∗*^ (PubChem ID: 5281139)	Same as no. 16	Same as no. 16	[124]
43	Caffeic acid^*∗*^ (PubChem ID: 689043)	Same as no. 17	Same as no. 17	[124]
44	Chlorogenic acid^*∗∗*^ (PubChem ID: 1794427)	Same as no. 15	Same as no. 15	[124]

I	Organosulfurs			

45	Onionin A	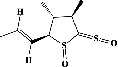	Antioxidant, antibacterial, cytotoxic, antitumoral, and antimetastatic activities	[166–169]
46	Cycloalliin (PubChem ID: 12305351)	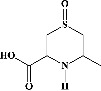	Antioxidant activity	[170, 171]
47	Isoalliin (PubChem ID: 5281112)	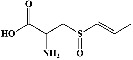	Antibacterial activity	[172, 173]
48	Methiin (PubChem ID: 9578071)	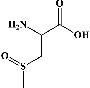	Antidiabetic and antioxidative activities	[172, 174]
49	Propiin (PubChem ID: 91819955)	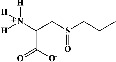	—	[172]
50	Allicin (PubChem ID: 65036)	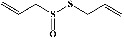	Antioxidant, antibacterial, antimalarial, and antimicrobial activities	[175–179]
51	Alliin (PubChem ID: 9576089)	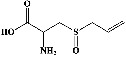	Antioxidant and antimicrobial activities	[172, 180–182]
52	S-methyl-L-cysteine (PubChem ID: 24417)	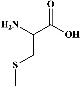	Antibacterial and antimicrobial activities	[172, 183–185]
53	S-ethylcysteine (PubChem ID: 25203803)	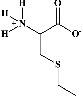	Antioxidative and antiglycative activities	[172, 186]
54	S-propyl-L-cysteine (PubChem ID: 125198)	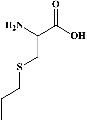	Antioxidant, antibacterial activities, and anticancer activities	[172, 187, 188]
55	S-allyl-cysteine (PubChem ID: 9793905)	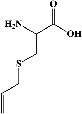	Antioxidant and anti-inflammatory activities	[172, 189, 190]
56	S-propylmercapto-cysteine (PubChem ID: 192762)	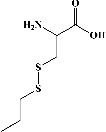	Antioxidant activity	[172, 191]
57	Trisulfide dipropyl (PubChem ID: 22383)	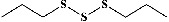	Antimicrobial activity	[172, 192]
58	Diallyl disulfide (PubChem ID: 16590)	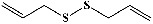	Antimicrobial and anticarcinogenic activities	[172, 193, 194]
59	Diisopropyl disulfide (PubChem ID: 77932)	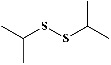	Antioxidative activity	[172, 195]
60	Methyl propenyl disulfide (PubChem ID: 5366552)	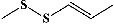	Antioxidant, antibacterial, and antimicrobial activities	[172, 196, 197]
61	6-Methyl-4,5-dithia-1-heptene (PubChem ID: 525503)	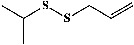	—	[172]

J	Anthocyanin			

62	Cyanidin 3-glucoside (PubChem ID: 12303220)	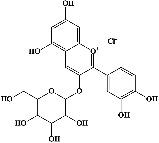	—	[198]
63	Cyanidin 3-laminaribioside (PubChem ID: 44256721)	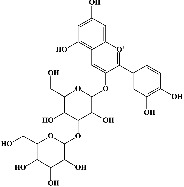	Antioxidant activity	[141, 199, 200]
64	Cyanidin 3-malonylglucoside (PubChem ID: 10143380)	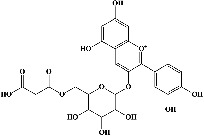	Antimicrobial and antiobesity activities	[141, 201–203]
65	Petunidin 3′-glucoside acetate (PubChem ID: 443651)	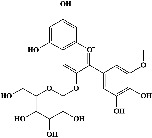	Antioxidant activity	[141, 204]
66	Malvidin 3′-glucoside (PubChem ID: 11249520)	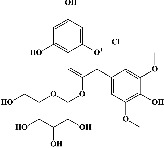	Antioxidant and anti-inflammatory activities	[141, 205, 206]
67	Peonidin-3′-glucoside (PubChem ID: 443654)	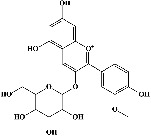	Anti-inflammatory and antioxidant activities	[141, 207, 208]

^*∗*^ = first-time repeated compound; ^*∗∗*^ = second-time repeated compound. PubChem IDs were taken from the PubChem website (https://pubchem.ncbi.nlm.nih.gov/) in order to get the 2D structure of compounds for molecular docking.

**Table 2:** The list of 28 compounds, their structures (along with rolipram, as standard), free bind energies (∆*G*_bind_ [kcal/mol]), and pIC_50_ values.

Sr. no.	Compounds	∆*G*_bind_ (kcal/mol)	pIC_50_
1	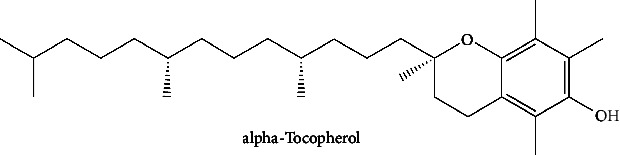	−38.007	25.46
2	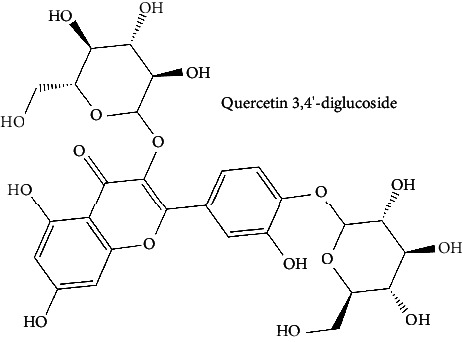	−29.557	10.9
3	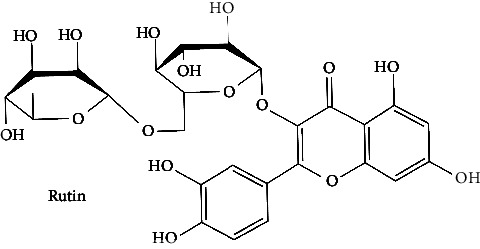	−25.637	8.23
4	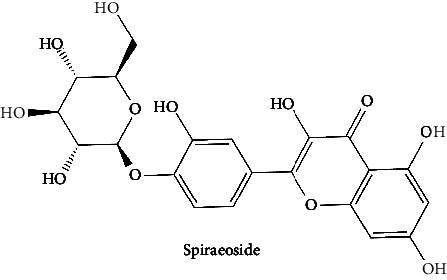	−25.318	7.78
5	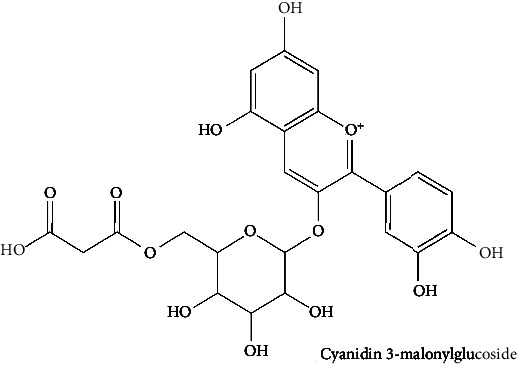	−24.354	6.44
6	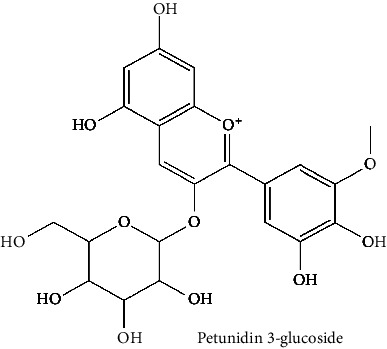	−24.036	5.99
7	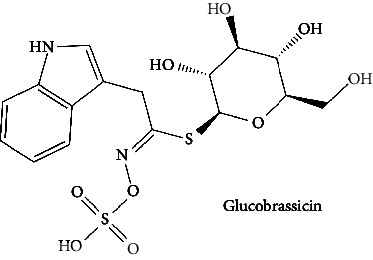	−23.865	5.7
8	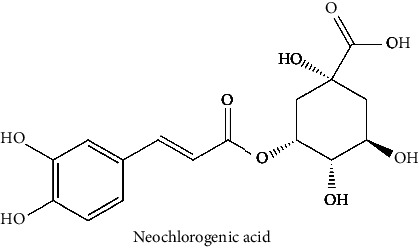	−23.789	5.6
9	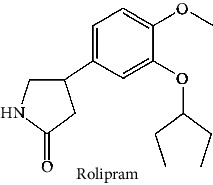	−22.801	4.27
10	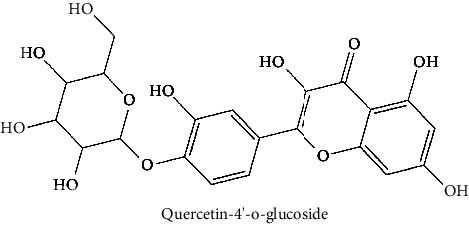	−21.833	2.93
11	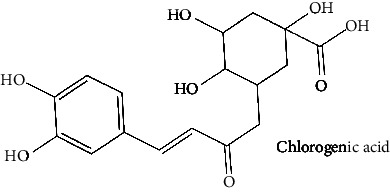	−21.501	2.5
12	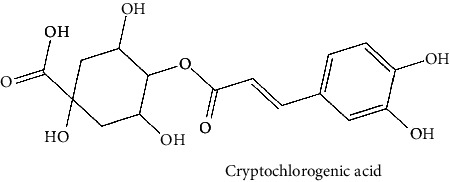	−21.479	2.43
13	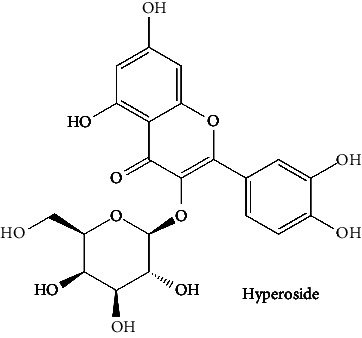	−21.169	2
14	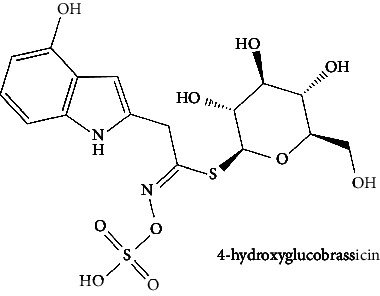	−20.423	0.96
15	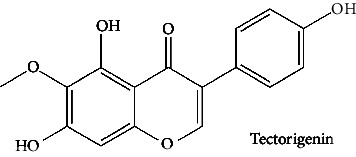	−19.686	−0.06
16	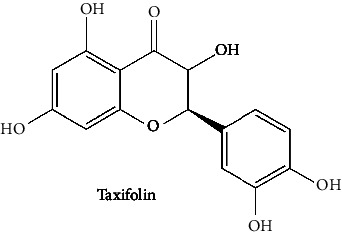	−18.621	−1.55
17	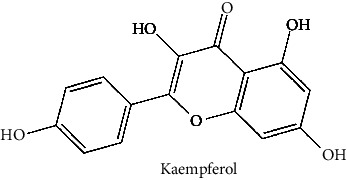	−17.410	−3.24
18	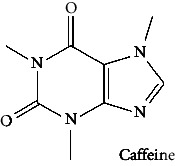	−17.287	−3.41
19	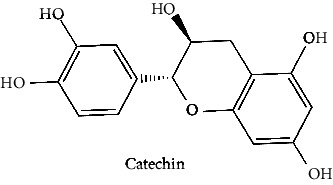	−17.271	−3.43
20	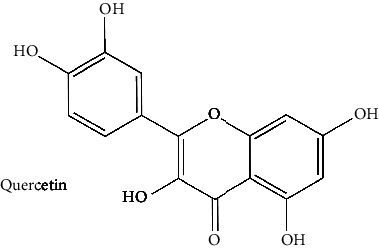	−17.252	−3.45
21	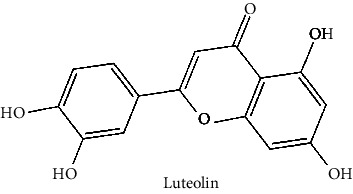	−16.574	−4.4
22	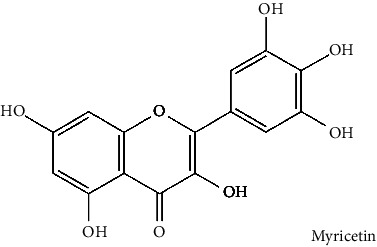	−16.461	−4.56
23	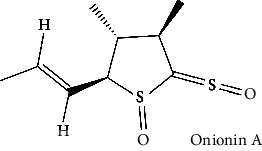	−15.621	−7.53
24	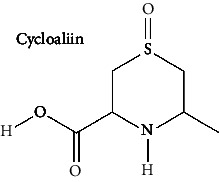	−14.850	−6.8
25	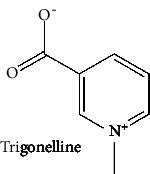	−14.099	−7.85
26	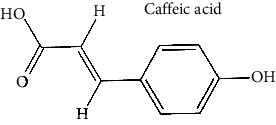	−13.874	−8.16
27	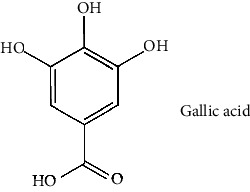	−11.439	−11.55
28	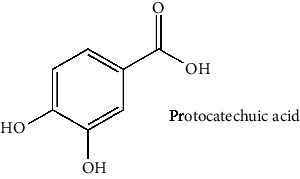	−10.316	−13.12

∆*G*_bind_ = free bind energy; kcal/mol = kilocalorie/molar; IC_50_ = half-maximal inhibitory concentration; pIC50 = −log(IC_50_ (M)).

**Table 3:** The computational pharmacokinetic and toxicities studies of 61 reported compounds derived from Swiss ADME and OSIRIS.

Compound	GI absorption^a^	Solubility^b^	BBB permeant^c^	PAINS^d^	Log Po/w^e^	Mutagenecity^f^	Tumorigenic^g^	Irritant^h^	Reproductive effective^i^	Pgp substrate	CYP1A2 inhibitor	CYP2C19 inhibitor	CYP2C9 inhibitor	CYP2D6 inhibitor	CYP3A4 inhibitor	Bioavailability score
1	Low	Very soluble	No	No	−1.26	No	No	No	Yes	Yes	No	No	No	No	No	0.11
2	Low	Very soluble	No	No	−1.54	No	No	No	Yes	Yes	No	No	No	No	No	0.11
3	Low	Soluble	No	No	−0.2	No	No	No	Yes	Yes	No	No	No	No	No	0.11
4	Low	Soluble	No	No	−0.45	No	No	No	Yes	No	No	No	No	No	No	0.11
5	Low	Very soluble	No	No	−0.94	No	No	Yes	Yes	Yes	No	No	No	No	No	0.11
6	Low	Soluble	No	No	−0.71	No	No	No	Yes	No	No	No	No	No	No	0.11
7	Low	Very soluble	No	No	−1.1	No	No	No	Yes	Yes	No	No	No	No	No	0.11
8	Low	Very soluble	No	No	−0.52	No	No	Yes	Yes	Yes	No	No	No	No	No	0.11
9	High	Soluble	No	Yes	1.23	Yes	Yes	No	No	No	Yes	No	No	Yes	Yes	0.55
10	High	Soluble	No	Yes	0.83	No	No	No	No	Yes	No	No	No	No	No	0.55
11	High	Soluble	No	No	1.58	Yes	No	No	No	No	Yes	No	No	Yes	Yes	0.55
12	High	Soluble	No	Yes	1.73	No	No	No	No	No	Yes	No	No	Yes	Yes	0.55
13	High	Soluble	No	Yes	0.32	—	—	—	—	Yes	Yes	No	No	No	No	0.55
14	Low	Very soluble	No	Yes	−0.39	No	No	No	No	No	No	No	No	No	No	0.11
15	High	Soluble	Yes	No	1.26	No	No	No	Yes	No	No	No	No	No	No	0.85
16	High	Very soluble	No	Yes	0.93	Yes	Yes	No	Yes	No	No	No	No	No	No	0.56
17	High	Very soluble	No	Yes	0.21	Yes	No	No	Yes	No	No	No	No	No	Yes	0.56
18	High	Highly soluble	No	No	−1.24	No	No	No	No	No	No	No	No	No	No	0.56
19	Low	Very soluble	No	Yes	−0.37	No	No	No	No	No	No	No	No	No	No	0.11
20	Low	Very soluble	No	Yes	−0.46	No	No	No	No	No	No	No	No	No	No	0.11
21	High	Very soluble	No	No	0	Yes	Yes	No	Yes	No	No	No	No	No	No	0.55
22	High	Very soluble	No	No	−0.61	No	No	No	No	No	No	No	No	No	No	0.55
23	Low	Soluble	No	No	−0.19	Yes	No	No	No	Yes	No	No	No	No	No	0.17
24	Low	Soluble	No	Yes	0.79	Yes	No	No	No	No	Yes	No	No	No	Yes	0.55
25	High	Soluble	No	Yes	0.51	No	No	No	No	No	No	No	No	No	No	0.55
26	Low	Soluble	No	Yes	1.3	Yes	No	No	No	No	Yes	No	No	Yes	Yes	0.55
27	High	Soluble	No	No	1.65	Yes	No	No	No	No	Yes	No	No	Yes	Yes	0.55
28	Low	Soluble	No	Yes	−0.38	No	No	No	No	No	No	No	No	No	No	0.17
29	Low	Soluble	No	No	−0.19	Yes	No	No	No	Yes	No	No	No	No	No	0.17
30	High	Soluble	No	No	2.06	No	No	No	Yes	No	Yes	No	No	Yes	Yes	0.55
31	High	Soluble	Yes	No	1.36	Yes	Yes	No	Yes	No	No	No	No	No	No	0.85
32	High	Very soluble	No	Yes	0.65	Yes	No	No	No	No	No	No	No	No	Yes	0.56
33	High	Very soluble	No	No	1.34	No	No	No	No	No	No	No	No	No	No	0.55
34	High	Highly soluble	No	No	−1.44	No	No	No	No	No	No	No	No	No	No	0.55
35	High	Highly soluble	No	No	−1.21	No	No	No	No	No	No	No	No	No	No	0.55
36	High	Highly soluble	No	No	−1.91	No	No	No	No	No	No	No	No	No	No	0.55
37	High	Highly soluble	No	No	−1.87	No	No	No	No	No	No	No	No	No	No	0.55
37	High	Very soluble	Yes	No	1.61	No	No	No	No	No	No	No	No	No	No	0.55
39	High	Highly soluble	No	No	−1.06	No	No	No	No	No	No	No	No	No	No	0.55
40	High	Highly soluble	No	No	−1.42	No	No	No	No	No	No	No	No	No	No	0.55
41	High	Highly soluble	No	No	−0.34	No	No	No	No	No	No	No	No	No	No	0.55
42	High	Highly soluble	No	No	−0.45	No	No	No	No	No	No	No	No	No	No	0.55
43	High	Very soluble	No	No	0.15	No	No	No	No	No	No	No	No	No	No	0.55
44	High	Soluble	Yes	No	2.98	No	No	No	No	No	No	No	No	No	No	0.55
45	High	Very soluble	Yes	No	2.39	No	No	No	No	No	No	No	No	No	No	0.55
46	High	Soluble	Yes	No	2.54	No	No	No	No	No	No	No	No	No	No	0.55
47	High	Very soluble	Yes	No	1.85	No	No	No	No	No	No	No	No	No	No	0.55
48	High	Very soluble	Yes	No	2.46	No	No	No	No	No	No	No	No	No	No	0.55
49	Low	Soluble	No	Yes	−1.99	No	No	No	No	No	No	No	No	No	No	0.17
50	Low	Soluble	No	Yes	−1.31	—	—	—	—	No	No	No	No	No	No	0.17
51	Low	Soluble	No	Yes	−0.95	—	—	—	—	No	No	No	No	No	No	0.17
52	Low	Soluble	No	No	−1.9	No	No	No	No	No	No	No	No	No	No	0.17
53	Low	Soluble	No	No	−0.69	—	—	—	—	No	No	No	No	No	No	0.17
54	High	Highly soluble	No	No	−1.33	No	No	No	No	No	No	No	No	No	No	0.55
55	—	—	—	—	—	No	No	No	No	—	—	—	—	—	—	—
56	—	—	—	—	—	No	No	No	No	—	—	—	—	—	—	—
57	—	—	—	—	—	No	No	No	No	—	—	—	—	—	—	—
58	—	—	—	—	—	No	No	No	No	—	—	—	—	—	—	—
59	—	—	—	—	—	Yes	No	No	No	—	—	—	—	—	—	—
60	—	—	—	—	—	—	—	—	—	—	—	—	—	—	—	—
61	—	—	—	—	—	No	No	No	No	—	—	—	—	—	—	—

^a^ = GI (gastrointestinal absorption), ^b^ = water solubility, ^c^ = BBB (blood-brain barrier) permeant, ^d^ = the potential for a particular molecule to act as PAINS and obstruct biological assay is predicted by PAINS (pan-assay interference compounds), ^e^ = consensus logP (lipophilicity): the mean of five forecasts made with various methods (value < 5), ^f^ = mutagenicity (OSIRIS Property Explorer was used to carry out the evaluation), ^g^ = tumorigenic (OSIRIS Property Explorer was used to carry out the evaluation), ^h^ = irritant (OSIRIS Property Explorer was used to carry out the evaluation), ^i^ = reproductive effective (OSIRIS Property Explorer was used to carry out the evaluation), compounds (1 = sinigrin, 2 = progoitrin, 3 = 4-methoxyglucobrassicin, 4 = glucobrassicin, 5 = gluconapin, 6 = 4-hydroxyglucobrassicin, 7 = glucoalyssin, 8 = glucobrassicanapin, 9 = quercetin, 10 = catechin, 11 = kaempferol, 12 = luteolin, 13 = cyanidin, 14 = chlorogenic acid, 15 = p-coumaric acid, 16 = caffeic acid, 17 = gallic acid, 18 = ascorbic acid, 19 = cryptochlorogenic acid, 20 = neochlorogenic acid, 21 = caffeine, 22 = trigonelline, 23 = quercetin-4′-o-glucoside, 24 = myricetin, 25 = taxifolin, 26 = laricitrin, 27 = isorhamnetin, 28 = hyperoside, 29 = spiraeoside, 30 = tectorigenin, 31 = ferulic acid, 32 = protocatechuic acid, 33 = onionin A, 34 = cycloalliin, 35 = isoalliin, 36 = methiin, 37 = propiin, 38 = allicin, 39 = S-methyl-L-cysteine, 40 = S-ethylcysteine, 41 = S-propyl-L-cysteine, 42 = S-allylcysteine, 43 = S-propylmercaptocysteine, 44 = dipropyl trisulfide, 45 = diallyl disulfide, 46 = diisopropyl disulfide, 47 = methyl propenyl disulfide, 48 = 6-methyl-4,5-dithia-1-heptene, 49 = cyanidin 3-glucoside, 50 = cyanidin 3-malonylglucoside, 51 = petunidin 3-glucoside, 52 = malvidin 3-glucoside, 53 = peonidin-3-glucoside, 54 = alliin, 55 = rutin, 56 = violaxanthin, 57 = alpha-tocopherol, 58 = neoxanthin, 59 = quercetin 3,4′-diglucoside, 60 = cyanidin 3-laminaribioside, and 61 = *β*-carotene).

**Table 4:** Summary of PDE4B1 inhibitory activities (IC_50_ values) of test compounds, extracts, and rolipram.

Sr. no.	Test sample	IC_50_ values (*µ*M)^*∗*^
1	Sinigrin	0.27 ± 0.009
2	Chlorogenic acid	0.20 ± 0.03
3	Quercetin	0.25 ± 0.01
4	Combo standard	0.17 ± 0.03
5	Coffee extract	0.18 ± 0.03
6	Red onion	0.22 ± 0.02
7	White cabbage extract	0.24 ± 0.01
8	Combo extract	0.12 ± 0.03
9	Rolipram	0.15 ± 0.008

^*∗*^The values were the average of the three independent experiments, expressed as means standard deviation (SD). IC_50_ = median inhibitory concentration and *µ*M = micromolar.

## Data Availability

The data used to support the findings of this study are included in the article.
